# Development and standardization of processing technique for ready-to-use lab fermented *Kanji* mix using refractance window dried black carrot powder

**DOI:** 10.1038/s41598-023-27450-5

**Published:** 2023-01-05

**Authors:** Preetinder Kaur, Ruchika Zalpouri, Ritika Modi, Param Pal Sahota, Tarsem Singh Dhillon, Amrit Kaur

**Affiliations:** 1 Department of Processing and Food Engineering, Punjab Agricultural University, Ludhiana, India; 2 Department of Microbiology, Punjab Agricultural University, Ludhiana, India; 3 Department of Vegetable Science, Punjab Agricultural University, Ludhiana, India; 4 Department of Mathematics, Statistics and Physics, Punjab Agricultural University, Ludhiana, India

**Keywords:** Microbiology, Engineering

## Abstract

Black carrots are rich in bio-actives but underutilized owing to their short-term availability and perishable nature. Traditionally, black carrots have been used for the preparation of Kanji—a fermented non-dairy beverage prepared using natural fermentation by lactic acid bacteria and a few spices. This plant-based probiotic beverage has high antioxidant properties but there is a risk of contamination with pathogens due to uncontrolled fermentation during storage. To enhance the availability of this nutritious beverage throughout the year and to ensure the microbiological safety of the traditional fermented product, the present study was planned to optimize the process for controlled fermentation using freeze-dried lactic acid bacterial (LAB) culture and refractance window-dried black carrot powder. The physicochemical and microbiological profiles of LAB-fermented Kanji were analysed. The dried Kanji mix can be reconstituted into naturally fermented probiotic beverage with unique flavour and aroma along with ensured microbiological safety and enhanced commercial value.

## Introduction

Consumer demand for functional non-dairy products has risen and probiotics are used to create ready-to-drink drinks made from fruits and vegetables. Fermented foods with plant origin have been evaluated as vectors for administration of probiotic lactic acid bacterial cultures following the proficiency of the production of vegetable based fermented products via lactic acid bacteria^1^. Further, the likeness of consumers towards fresh like, minimally processed, highly nutritive, health-promoting and thirst quenching ready-to-serve beverages is increasing^2^. Traditionally, food fermentation using lactic acid bacteria is being performed since time immemorial and the utilization of functional starter cultures improve the functional quality of the products.

Black carrots (*Daucus carota* subsp. *sativus*) are in greater consumer’s interest due to their rich antioxidant, anthocyanins content and other phytochemicals content^3,4^. However, poor post-harvest management and delayed marketing significantly alter the concentrations of its bioactive compounds^5,6^. Fermented and probiotic black carrot beverage is a healthy option due to its high antioxidant activity aiding against lifestyle diseases and chronic ailments properties including improvement of lactose metabolism, prevention of gut infections, enhancement of immunity, reduction in serum cholesterol level, stimulation in calcium absorption, synthesis of vitamins (vitamin-B, folic acid and nicotinic acid), enhancement of protein digestibility and counteraction against ill effects of food borne pathogens^7^.

Traditional lactic acid fermented ready-to-serve black carrot beverage, commonly known as ‘*Kanji’* and is helpful in treatment of indigestion, loss of appetite and liver disorders. During traditional fermentation of black carrots, natural lactic acid fermentation imparts probiotic properties to the minimally processed beverages but risk of contamination with pathogens *Cronobacter sakazakii*, *Klebsiella pneumonia* and *Enterobacter hormaechei* has been detected in the stored naturally fermented black carrot beverage ‘*Kanji’*^8^. Hence, the need arises to ensure the microbiological safety of the traditional fermented product by using controlled fermentation techniques and aseptic handling for the large-scale production.

Controlled fermentation conditions utilizing pure function lactic acid starter culture result in enhanced control over the fermentation process and product uniformity. However, the high nutritional value of fermented *Kanji* lends itself to the growth of several spoilage microbes during storage through longer duration. As a result, it is not available for consumption during off seasons. The availability of this nutritional beverage can be enhanced muti-fold if it is made shelf stable which is possible through advanced drying techniques. Although many techniques for drying of fruits/ vegetables are in practice but some are incapable of preserving the bioactive compounds like open sun drying, solar drying and hot-air drying, etc.^9,10^. While the drying techniques that can preserve bioactives are quite expensive like freeze drying.

Refractance window drying (RWD) is a new and revolutionary process developed by MCD Technologies Inc (Tacoma, Washington, USA)^11,12^. The RWD approach has been shown to be effective for drying heat-sensitive materials such as liquids and purees into powders, flakes, or sheets^13^. This drying technique outperforms the conventional method due to reduced drying temperature, drying time, energy consumption, and greater quality retention. The researchers have found that results of RW dried potato^14^, carrot^15^, tomato^16^, goldenberry^17^ and aloe vera^18^ had maximum quality retention. Considering the advantage of RWD technique, the present study was planned to dry black carrot puree using RW dryer then fermented with freeze dried LAB to produce reconstituted *Kanji*.

## Material and methods

### Optimization of process parameter for producing RW-dried black carrot powder

#### Preparation of sample

Fresh black carrots (cv. Punjab Black Beauty) were harvested in February were obtained from the Department of Vegetable Science at the Punjab Agricultural University (PAU) in Ludhiana, Punjab, India. Before use, black carrots were precooled in a walk-in cold room at 10 °C and 85% relative humidity. Experiments were carried out at the laboratories of Department of Processing and Food Engineering, Punjab Agricultural University (PAU), Ludhiana, India to achieve the desired result. For experimentation, the precooled black carrots were washed, peeled using hand peeler and top of carrots were removed. The peeled black carrots were treated by dipping in sodium hydroxide (NaOH) solution at three different concentrations (i.e., 0, 1 and 2% v/v). The treated samples were then processed into puree using pulper. The freshly prepared puree was dried using batch type pilot scale refractance.

#### Experimental design, optimization and statistical analysis

The RSM was used to assess the effect of two independent variables, namely water temperature (x_1_) and NaOH concentration (x_2_), on several quality metrics such as colour change, total flavonoid content, total phenolic content, and rehydration ratio. Experiments were conducted in duplicate. Based on a two-factor three-level miscellaneous experimental design, thirteen experimental runs were developed. The experiment was randomized, with the centre point repeated five times. ANOVA and regression surface analysis were utilised to describe the statistical significance of model variables and to fit a regression connection appropriate to the experimental design. The terms that were statistically significant (*p* < 0.05) were included in the final model^19^. The following is the generalised polynomial model developed to predict response variables based on independent factors:

$$y_{k} \, = \, \beta_{0} \, + \, \sum\limits_{{{\text{i}} = {1}}}^{{\text{n}}} {\beta_{i} x_{i} \, + \, \sum\limits_{{{\text{i}} = {1}}}^{{\text{n}}} {\beta_{ii} x_{i}^{{2}} + \, \sum\limits_{{{\text{i}} = {1}}}^{{\text{n - 1}}} {} \sum\limits_{{{\text{ j}} = i + {1}}}^{{\text{n}}} {\beta_{ij} x_{i} x_{j} \, } } }$$

where y_k_ is the model's projected response value; *β*_0_, *β*_i_, *β*_ii_, and *β*_ij_ are constants, regression coefficients for linear, quadratic, and interaction effects factors, and xi is the coded independent variable. The Miscellaneous 3-Level Factorial in Design expert programme (Version 7.0.0) was used for the experimental design and data analysis (Stat-Ease Inc., Minneapolis, MN, USA). The response surface and contour plots were created for various interactions. These three-dimensional surfaces might give a precise geometrical representation as well as important information on the system's behaviour within the experimental design. The RW-dried black powder was tuned to determine the quantities of independent components that would result in the least degree of colour change and the highest total flavonoid content, total phenolic content and rehydration ratio.

### Process standardization for preparation of LAB fermented *Kanji* prepared from RW-dried black carrot powder

The optimized dried sample obtained based on minimum colour change and maximum total flavonoid content, total phenolic content and rehydration ratio was used in preparation of probiotic black carrot drink (*Kanji*).

#### Lactic acid bacterial cultures (production and lyophilization)

Functional Lactic Acid Bacteria as starter culture for preparation of traditional drink *Kanji* from dried black carrots powder were procured from Department of Microbiology, PAU, Ludhiana, India. A 24-h cultivation of consortium of ten LAB in the MRS broth was carried out at 37 °C, for obtaining a cell density of 10^7^ cfu/ml (before lyophilization). Cells were extracted by centrifuging at 11,000 × *g* for 5 min at 4 °C, followed by three washes with sterile distilled water. The supernatant was then discarded, and the captured cells were re-suspended in 100 mL of medium (final viable counts in the range of 7–8 log CFU/mL). Before lyophilization, all re-suspended cells were frozen at – 20 °C. The frozen suspensions were freeze-dried for 48 h (temperature − 40 ± 2 °C; vacuum pressure 10^−1^ torr) using a bench-top lyophilizer (Modulyo bench top freeze dryer, Edwards, Burgess Hill, UK). The retention of the microbial load in the freeze-dried culture (0.5–0.8% w/v) was using standard protocol. Lactic acid bacterial count equivalent to 7.9–8.96 log cfu/g makes an ideal starter culture for production of ready-to-use lactic acid fermented *Kanji*.

#### Optimization of ready-to-use LAB fermented *Kanji* mix

The mix was prepared using different loads of freeze-dried LAB and different concentrations of optimized RW-dried black carrot powder along with the standard spices to attain the desired *Kanji* formulation upon reconstitution. The reconstituted *Kanji* formulations were analysed for quality using standard procedures. The *Kanji* mix was optimized using RSM based upon the responses of designed experiment. The quality of reconstituted *Kanji* drink prepared under optimized conditions was compared with control *Kanji* drink prepared by traditional method using fresh black carrots to determine the acceptability of the developed ready to use *Kanji* mix.

#### Standardization of reconstitution process for LAB fermented *Kanji* mix

Rehydration standardization of RW-dried black carrot powder in water for getting the same desired traditional *Kanji* beverage taste was done using different proportions of powder with water 10:50, 10:60 and 10:70, on the basis of physicochemical characteristics, microbiological analysis and sensory evaluation.

### Traditional *Kanji* formulation and preparation

The fermentation of traditional *Kanji* beverage was carried out by autochthonous lactic acid bacteria inherent to black carrots, by extracting the carrot juice and shredded carrots and diluting it with three volumes of boiled and then cooled at ambient temperature water and allowed to ferment at room temperature (25 ± 2 °C) for 5 days with the addition of salt and rye at 1.5%. *Kanji* beverage preparation through controlled fermentation was carried with diluted beverage pasteurized at 82 °C for 10–15 s using pasteurizer (Dairy Tech, Maharashtra, India) and inoculating with 0.5% (v/v) of actively grown functional starter inoculum (10^8^ cfu mL^−1^) of functional lactic acid bacterial consortium. Other spices: Pink Rock Salt (1.5%), Rye (1.5%) were sterilized before use in the mix. A portion of pasteurized beverage was kept as control of the fermentation process. The inoculated beverage was incubated at 37 °C for 24–36 h (conditions determined in preliminary tests). Samples were aseptically drawn and analysed for microbiological and physicochemical parameters.

### Physicochemical, microbiological and sensory evaluation of dried black carrot powder and *Kanji* beverage

For RW-dried black carrot powder quality was measured on the basis of colour change, total flavonoid content, total phenolic content, and rehydration ratio. While quality for LAB fermented *Kanji* beverage and traditional *Kanji* beverage was measured on the basis of titratable acidity, pH, Brix-Acid ratio, total sugars, total reducing sugars, antioxidant activity, total flavonoid content, total phenol content, total carotenoids, ascorbic acid content. The following methods of each parameter have been explained below in various subheadings:

#### Colour change

A portable colorimeter (Konica Minolta Sensing Inc, Japan) was used to determine the colour of the samples^20^. The colour is defined by the tristimulus values L, a, and b. The colour change was determined using the following relationship^21^:

$${\text{Colour}}\;{\text{change}},\quad \Delta {\text{E}} = \sqrt {\left[ {\left( {{\text{L}} - {\text{L}}_{{\text{o}}} } \right)^{{2}} + \left( {{\text{a}} - {\text{a}}_{{\text{o}}} } \right)^{{2}} + \left( {{\text{b}} - {\text{b}}_{{\text{o}}} } \right)^{{2}} } \right]}$$

where, L_o_, a_o_ and b_o_ represent the respective readings of fresh black carrot puree.

#### Rehydration ratio

At 95 °C, the samples were submerged in water at a 1:15 ratio for 20 min. The excess water was removed using the Whatman paper no. 1, and the samples were weighed^22^.

#### pH

The pH of rehydrated *Kanji* beverage was evaluated using a digital pH metre (type 101, Electronic Corporation of India Limited, Hyderabad).

#### Total soluble solids (TSS)

Total soluble solids (TSS) in rehydrated carrot juice and beverage were examined by Erma hand Refractometer of 0–32°Brix (UNICO). The refractometer was calibrated at zero line on the scale by placing a drop of distilled water on clean and dry prism at 20 °C. The TSS value of samples was determined by adding a drop of sample on the clean prism and reading the clear line of demarcation on the scale.

#### Titratable acidity

Titratable acidity, expressed as %Lactic acid, was estimated following the procedure of Helrich^23^. Known quantity of beverage was used and few drops of 1% Phenolphthalein solution as indicator were added. Titration of solution was performed against standardized 0.1 N Sodium Hydroxide (NaOH) to the pink coloured end point persisted for 15 s.

#### Brix-acid ratio

Brix-Acid ratio was calculated by dividing Total soluble solids value with total acidity of the rehydrated juice and beverage to evaluate the ripeness of carrots.

#### Total sugars

Total sugars were determined using the DuBois et al.^24^ technique. In the test tubes, measured volumes of 0.1–0.5 ml of sample/standard were placed, and the volume was increased to 1 mL using distilled water. Each test tube received 1 ml of a 5 percent phenol solution, which was well shaken. Following that, 5 ml of strong sulphuric acid was added. For the best appearance of yellow colouring, sulfuric acid was poured straight into the centre of the test tube and the temperature was raised to 70 °C. The test tubes were left at room temperature for 10 min before being cooled. The absorbance of the produced stable yellow-orange colour was measured at 490 nm with a spectrophotometer (Bausch & Laumb Spectronic-20) against a reagent blank. The total soluble sugars were determined using glucose as the standard curve (20–100 mg mL^−1^).

#### Total reducing sugars

Miller^25^ method was used for the quantification of total reducing sugars in the samples. 3 mL of the sample and 3 mL of DNS reagent were added in the test tube and kept in the hot water bath at 60 °C for 15 min. 1 ml Rochelle salt solution was then added to the tubes and were allowed to cool down. The absorbance was taken at 575 nm using spectrophotometer (Bausch & Laumb Spectronic-20). The standard curve was used to calculate the concentration of reducing sugars (20–100 g mL^−1^).

#### Antioxidant activity (%) by DPPH assay

DPPH technique given by de Ancos et al.^26^ was used to calculate the percent antioxidant activity. Aliquots of 0.1 ml sample were collected in a test tube for estimation. It was placed in the dark for 45 min after the addition of 3.9 mL DPPH solution (1 M DPPH). The discoloration in the solution was measured with a Bausch & Laumb Spectronic-20 at 515 nm.

#### Total flavonoid content

Total flavonoid content was evaluated by the method given by Carvalho and Clemente^27^ with small modifications. In a test tube, 0.2 ml of each standard or sample was placed, then 1 mL of methanol was added. 1 ml of methanol was used to make the blank. Each test tube received 0.1 mL of a 10% aluminium nitrate solution. After that, 0.1 mL of 1 M potassium acetate was added, followed by 4.6 ml of distilled water. The materials were properly combined and left at room temperature for 45 min. The absorbance was measured at 415 nm against a methanol reference blank. The total flavonoid content (mg of Quercetin equivalents) was estimated using the Quercetin standard curve.

#### Total phenolic content

The total phenolic content was determined using the technique of Slinkard and Singleton^28^, with minor modifications. 0.2 ml of each sample/standard was placed in a test tube, and a volume of 1 ml was produced with methanol. The materials were well mixed after 2 ml of Folin-ciocalteu reagent was added, then 2 mL of 15% Na_2_CO_3_ solution was added after four minutes. The mixture was maintained at room temperature for two hours. The absorbance of each sample was taken at 760 nm against a reference was measured at 760 nm against a blank prepared in the similar way as of the samples without the addition of sample or standard. Total phenolic content (mg Gallic acid equivalents) was calculated from standard curve of Gallic acid.

#### Ascorbic acid content

The titrimetric method with 2, 6- Dichlorophenol Indophenol dye was used in the estimation of ascorbic acid^29^. Measured volume of an aliquot (10 ml) was taken and 15 ml of oxalic acid solution (0.4%) was added followed by titration against standardized dye solution (0.04%) to a pink colour end point with 15 s of persistence.

#### Total carotenoids

Carotenoids extraction was based on the procedure described by Rodriguez-Amaya and Kimura^30^. 5 ml of each sample was homogenized using chilled acetone and filtered using a Büchner funnel. The technique was repeated until the residue and pigments were discoloured and transferred to petroleum ether; each fraction was rinsed with distilled water to remove all traces of acetone. After full extraction, the total carotenoids content of the isolated pigments was measured spectrophotometrically at 450 nm.

#### Viability of LAB in *Kanji* beverage

The viability of bacteria in rehydrated *Kanji* beverage was evaluated by standard plate count method. Bacterial plate count was recorded after 24–48 h at 37 °C on Man Ragosa Sharpe Agar (HiMedia Laboratories Pvt Ltd, Mumbai) for lactic acid bacterial count^8^.

#### Sensory evaluation of *Kanji* beverage

The 30 ml LAB fermented *Kanji* beverages were placed separately in labelled plastic cups. These cups were kept at room temperature (28 ± 3 °C) inside cubical having daylight settings. Thirty panellists (15 male and 15 female) were recruited from staff and students at the University of Punjab Agricultural University. Drinking water was provided for subjects to rinse their mouth between two drinks and five minutes time was given between each drink. A semi-trained panel of judges evaluated the organoleptic properties of black carrot beverage based on appearance, taste, colour, aroma, body, flavour, astringency, and overall acceptability. The "Hedonic scale" of consumer acceptability was used to calculate product acceptance^31^.

## Result and discussion

### Optimization of process variables

**Figure 1:**
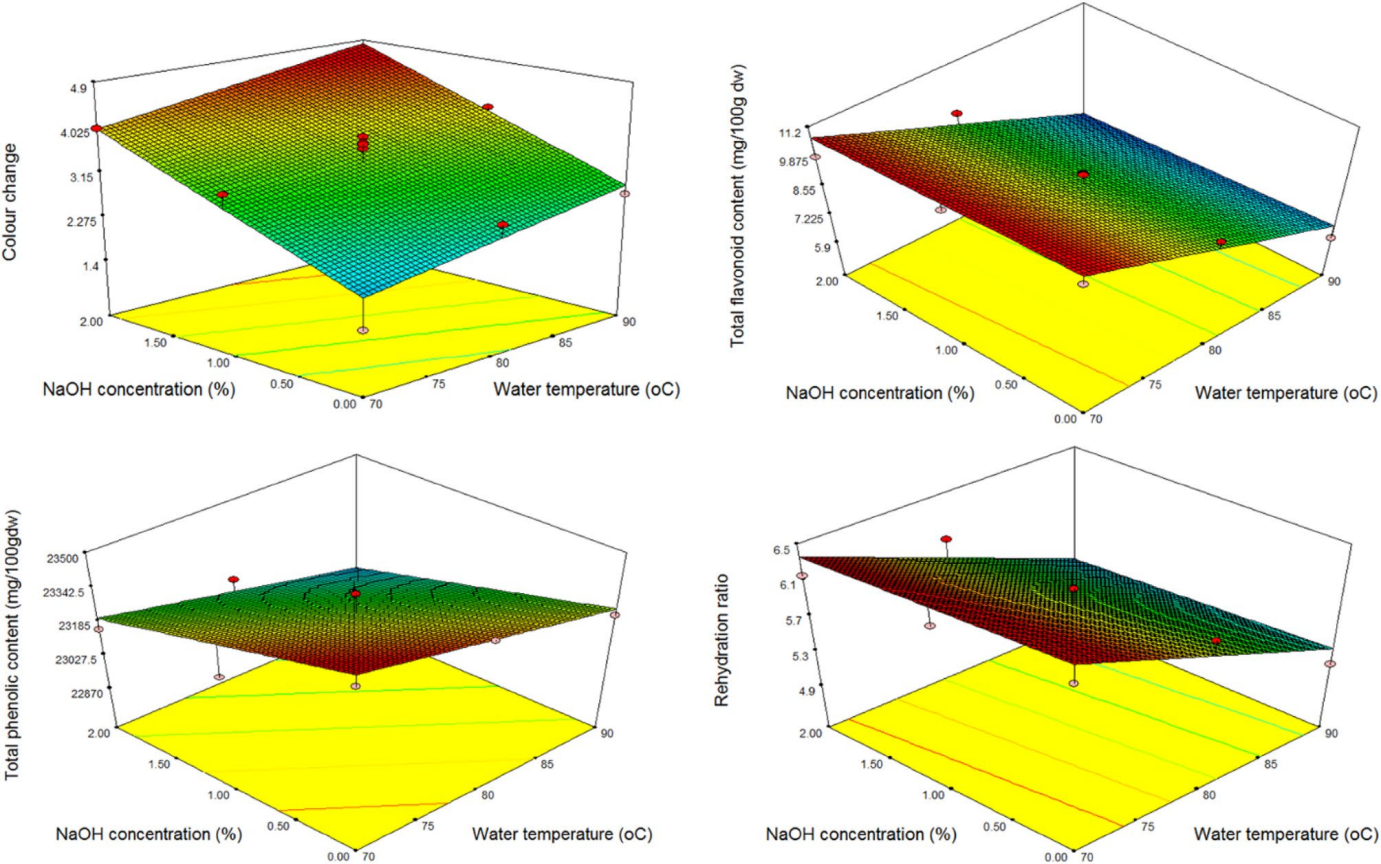
Effect of water temperature and NaOH concentration on quality parameters of dried black carrot.

The RW-dried black carrot powder was produced by adjusting two process variables, namely water temperature (70, 80, and 90 °C) and NaOH solution concentration (0, 1, and 2% v/v), and the method was adjusted based on the impacts on chosen responses (colour change, flavonoids, total phenolic content and rehydration ratio). Table 1 shows the analysis of variance for all responses, and three-dimensional response surface plots (Fig. 1) were created to show the influence of process factors on the response parameters of the RW-dried black carrot powder.

**Table 1:** Regression summary and ANOVA for quality parameters for RW-dried black carrot puree.

Source	Colour change			Total flavonoid content			Total phenolic content			Rehydration ratio		
	Sum of Squares	F-value	*p*-value	Sum of Squares	F-value	*p*-value	Sum of Squares	F-value	*p*-value	Sum of Squares	F-value	*p*-value
Model	6.571	29.246	< 0.0001*	29.253	50.588	< 0.0001*	211,082.010	11.024	0.003*	2.021	22.954	0.001*
Water temperature	1.042	9.272	0.012*	29.040	100.440	< 0.0001*	88,139.064	9.206	0.013*	1.995	45.331	< 0.0001*
NaOH concentration	5.530	49.220	< 0.0001*	0.213	0.736	0.411	2942.946	0.842	0.715	0.025	0.576	0.465
Lack of Fit	0.792	1.590	0.340	2.888	2.012	0.322	4686.763	0.321	0.564	0.440	0.987	0.476
R-Squared	–	0.854	–	–	0.910	–	–	0.888	–	–	0.911	–
Adj R-Squared	–	0.825	–	–	0.892	–	–	0.826	–	–	0.885	–

### Physicochemical quality of black carrot powder

#### Colour change

Colour is a key quality factor that is used to assess the quality of any food product. As a result, changes in colour aid in identifying changes in product quality^32^. The colour change of dried black carrot ranged from 1.48 to 4.61. The maximum colour change was observed for samples pre-treated in 2%NaOH solution and dried at 90 °C water temperature whereas minimum was found for untreated samples dried at 70 °C. The linear term of water temperature and NaOH concentration had significant positive effect on colour change (*p* < 0.05). It was observed that with increase in NaOH concentration during pre-treatment of black carrot had increased the colour change in black carrot powder (Fig. 1). This was due to increase in ‘L’ value of carrot, hence increasing overall colour change value when compared with fresh produce. The rise in water temperature also resulted in an increase in colour change, which may be ascribed to an increase in ‘a’ value than the ‘b’ value of black carrot powder^33^. The increase in ‘a’ value has been reported in various foods owing to chemical reaction that occurs between amino acids and reducing sugars while processing at higher temperatures which generate brown colour and subsequent occurrence of Milliard’s reaction^34^.

$${\text{Colour}}\;{\text{change}}\;{\text{of}}\;{\text{black}}\;{\text{carrot}}\;{\text{powder}} = + 3.46 + 0.42*{\text{water}}\;{\text{temperature}} + 0.96*{\text{NaOH}}\;{\text{concentration}}$$

#### Total flavonoid content (mg/100 g dw)

Flavonoids have been demonstrated to be helpful to human health due to their capacity to serve as free radical scavengers and oxidative potential-reducing agents, hence protecting against oxidative damage produced by hydroxyl groups^35^. The total flavonoid content of dried black carrot ranged from 5.97–10.75 mg/100 g dw. The minimum total flavonoid content was observed for samples pre-treated in 2%NaOH solution and dried at 90 °C water temperature whereas maximum was found for untreated samples dried at 70 °C. The linear term of water temperature had significant negative effect on total flavonoid content (*p* < 0.05). It was discovered that the total flavonoid content of black carrot powder decreased with an increase in water temperature (Fig. 1). This might be because higher temperatures caused partial lignin breakdown and thermal degradation of phenolic compounds^36^.

$${\text{Total}}\;{\text{flavonoid}}\;{\text{content}} = + 8.72 - 2.20*{\text{water}}\;{\text{temperature}} - 0.19*{\text{NaOH}}\;{\text{concentration}}$$

#### Total phenolic content (mg/100 g dw)

Total phenolic content of dried black carrot ranged from 22,876.40 to 23,444.30 mg/100gdw. The maximum colour change was observed for untreated samples dried at 70 °C minimum was found for samples whereas pre-treated in 2%NaOH solution and dried at 90 °C water temperature. The linear term of water temperature had significant negative effect on total phenolic content (*p* < 0.05). It was observed that with increase in water temperature of dryer decreased the total phenolic content in black carrot powder (Fig. 1). One such explanation for the decrease in overall phenolic content at high temperatures is the impact of heat on tannic compounds and the death of cells and their vacuoles, which causes phenolic compounds to attach to other compounds such as proteins or changes in their chemical structures^18^.

$${\text{Total}}\;{\text{phenolic}}\;{\text{content}} = + 23229.12 - 121.20*{\text{water}}\;{\text{temperature}} - 143.15*{\text{NaOH}}\;{\text{concentration}}$$

#### Rehydration ratio

Rehydration ratio of dried black carrot ranged from 4.99 to 6.30. The minimum rehydration ratio was observed for samples pre-treated in 2%NaOH solution and dried at 90 °C water temperature whereas maximum was found for untreated samples dried at 70 °C. The linear term of water temperature had significant positive effect on rehydration ratio (*p* < 0.05). The rehydration ratio of black carrot powder was found to decrease with an increase in the water temperature of dryer. (Fig. 1). This can be owing to overheating of the product surface causing development of an impervious layer making it difficult for the dried product to rehydrate to its actual volume and shape^33,37^.

$${\text{Rehydration}}\;{\text{ratio}} = + {5}.{85} - 0.{58}*{\text{water}}\;{\text{temperature}} - 0.0{65}*{\text{NaOH}}\;{\text{concentration}}$$

### Optimization of RW-dried black carrot powder

Using the numerical optimization technique, the optimum process conditions for black carrot powder was obtained by dipping black carrot in 0% NaOH solution followed by refractance window drying at water temperature of 70 °C. Black carrot powder produced under optimized condition predicted to have the highest value of total flavonoid content: 11.11 mg/100 g dw, total phenolic content: 23,493.50 mg/100g dw and rehydration ratio: 6.49 whereas the least possible colour change: 2.08 with overall desirability of 94.8%. The samples were further dried in bulk under the optimized process condition to produce black carrot powder for reconstituted *Kanji* beverage.

### Standardization of RW-dried black powder reconstitution ratio for *Kanji* beverage formulation and fermentation

For the evaluation of the right reconstitution ratio of RW-dried black carrot powder for beverage formulation, the three proportions of powder with water i.e., 10:50, 10:60 and 10:70 were prepared and subjected to sensory evaluation. The sensory evaluation was carried out on a 9-point Hedonic scale. The score obtained from the panellists has been given in the Table 2. From the sensory scores, it is clearly observed that the 10:60 combination of RW-dried black carrot powder and water have shown better acceptability (7.8 ± 0.2) compared to other combinations.

**Table 2:** Sensory evaluation of different rehydration volumes of water for lactic acid bacterial fermentation of RW-dried *Kanji* powder.

Sensory attributes	10:50 reconstitution ratio	10:60 reconstitution ratio	10:70 reconstitution ratio
Appearance	8.5 ± 0.2ab	8.6 ± 0.3a	8.0 ± 0.2b
Taste	6.0 ± 0.1c	7.5 ± 0.2a	6.4 ± 0.3b
Color	7.8 ± 0.3c	8.4 ± 0.2a	8.2 ± 0.1b
Aroma	6.0 ± 0.2c	8.1 ± 0.1a	6.5 ± 0.1b
Body	6.2 ± 0.3b	7.8 ± 0.2a	6.5 ± 0.3b
Flavor	6.8 ± 0.2b	8.2 ± 0.2a	6.5 ± 0.1c
Astringency	5.8 ± 0.3c	7.5 ± 0.1a	6.0 ± 0.2b
Overall acceptability	6.4 ± 0.1c	7.8 ± 0.2a	6.8 ± 0.1b

### Standardization of functional freeze-dried LAB inoculum concentration in *Kanji* beverage for fermentation

Functional lactic acid bacterial inoculum concentration, containing the cell load of 10^7^ cfu/ml was used as inoculum in developing lactic acid fermented *Kanji* beverage. The varied percentages of lyophilized inoculum concentration were taken (0.4–0.8% w/v) and incorporated in the standardized RW-dried *Kanji* beverage reconstituted (10:60) and fermented for 24 h at 37 °C to see the variation in cell load with respect to the lactic acid bacterial cell load in the fermented traditional beverage. The data obtained after enumeration studies in traditionally fermented and after freeze drying fermentation have been depicted in Table 3. Functional lactic acid bacterial count in RW-dried black carrot powder fermented with 0.7% (w/v) freeze-dried inoculum was in line with cell load in traditionally fermented *Kanji* beverage. Hence, a minimum of 0.7% (w/v) freeze-dried inoculum concentration is required that fulfils the requirements in traditionally fermented *Kanji* beverage.

**Table 3:** Functional freeze-dried LAB inoculum concentration optimization for *Kanji* beverage fermentation.

Inoculum concentration	Cell load before fermentation (Log cfu/ml)	Cell load after fermentation (Log cfu/ml)
Traditional *Kanji* beverage	7.94 ± 0.01 c	9.14 ± 0.01 c
FD 0.4% (w/v)	6.24 ± 0.01 f	7.98 ± 0.01 f
FD 0.5% (w/v)	7.54 ± 0.02 e	8.27 ± 0.01 e
FD 0.6% (w/v)	7.91 ± 0.01 d	8.97 ± 0.01 d
FD 0.7% (w/v)	8.10 ± 0.02 b	9.21 ± 0.01 b
FD 0.8% (w/v)	8.64 ± 0.01a	9.48 ± 0.01 a

FD- freeze-dried inoculum concentration, mean ± standard deviation, similar letters have no significant effect.

### The microbiological and physicochemical analysis process parameters during fermentation

**Figure 2:**
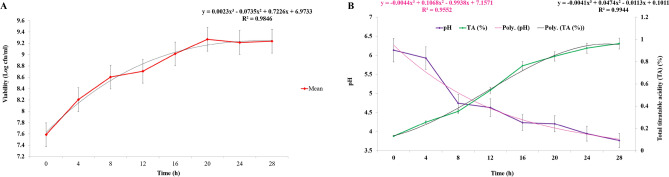
(**A**) The amount of microbial (lactic acid bacteria), (**B**) pH and total titratable acidity during LAB fermentation of *Kanji* beverage reconstituted at 10:60 concentration.

The black carrot powder was reconstituted according to the standardization with water and fermented with standardized freeze-dried 0.7% (w/v) inoculum concentration with a cell load of 9.21 log cfu/ml at 37 °C for 28 h and fermentation kinetics was studied in terms of lactic acid bacterial growth during the fermentation, pH and total titratable acidity kinetics. The proliferation of lactic acid bacteria during fermentation can provide vital information about the properties of fermented *Kanji* beverage. Figure 2A depicts the change in the quantity of lactic acid bacteria. The bacteria adapted quickly in the reconstituted beverage, resulting in an increase in viability from the original fermentation. The number of lactic acid bacteria in the beverage rapidly grew within the first 24 h, which corresponds to the usual microbial development curve. After the first 24 h, the number of lactic acid bacteria decreased slightly and then stabilised. Also, Fig. 2A showed that Polynomial 3rd order model showed best fit model to describe increase in viability of bacteria with highest R^2^ value of 0.985. Costa et al.^38^ discovered that after 24 h of fermentation by *L. casei* NRRL B-442, the viability in pineapple juice reached 8.65 log cfu/ml. After the first 24 h, the build-up of significant lactic acid and other metabolites impeded the proliferation of lactic acid bacteria. In brief, RWD-dried black carrot powder reconstituted with water (10:60 ratio) is an appropriate substrate for probiotics and can be used as a nutritional media to enhance LAB development.

Figure 2B depicts the results of an investigation into the pH and titratable acidity dynamics of fermented beverages. Because of the increasing titratable acidity content, the pH fell during the fermentation. After 12 h of fermentation, the pH of the *Kanji* beverage was lower than 4.0, which was a threshold for inhibiting pathogenic and numerous septic bacteria^38^. In Fig. 2B, Polynomial 3rd order model showed best fit model to describe decrease in pH and increase in TA with respect to time, having highest R^2^ value of 0.955 and 0.994 for pH and TA, respectively. After 28 h of fermentation, the titratable acidity rose from 0.13 g/100 ml to 0.96 g/100 ml. A similar rise was observed during fermentation of rice-based beverage by Ghosh et al.^39^. The increasing concentration of organic acids produced by the lactic acid bacteria fermentation process, particularly the formation of lactic acid, might explain the variations in pH and titratable acidity^7^.

The total phenolic content, total flavonoid content and antioxidant capacity of fermented beverage after 24 h of fermentation as compared to the initial 0 h shown in the Table 4. Phytochemical concentrations rise after 24 h as compared to the first stage, indicating that LAB enhanced the phytochemical concentration of the beverage during the fermentation process. The higher amount of phytochemical concentrations in fermented beverage might be ascribed to LAB's capacity to create hydrolytic enzymes, which hydrolyzed the complex phytochemicals into simpler forms^31,40^. The total phenolic content of the 24 h fermented samples increased from 32.73 ± 0.61 mg (at 0 h) to 41.84 ± 0.10 mg GAE/ml. Total phenolic content are widespread secondary metabolites of plants that are involved in plant defence against UV radiation or pathogen aggressiveness. Epidemiological research and accompanying meta-analyses clearly suggest that long-term intake of plant polyphenol-rich diets protects against the development of malignancies, cardiovascular illnesses, diabetes, osteoporosis, and neurological disorders^41^. Furthermore, the LAB-fermented *Kanji* beverage's lower pH (3.76) stabilises the polyphenols as they auto-oxidize with increasing pH^8^.

**Table 4:** Total phenolic content, flavonoids, antioxidant activity and carotenoids during LAB fermentation of *Kanji* beverage reconstituted at 10:60 concentration.

Fermentation time (Hours)	Total phenolic content (mg/100 ml)	Total flavonoids content (mg/100 ml)	Antioxidant activity (%)	Total carotenoids (mg/100 ml)
0	32.73 ± 0.61c	31.10 ± 0.13c	80.59 ± 0.91c	33.44 ± 0.02d
4	39.59 ± 0.32b	40.90 ± 0.31b	84.92 ± 0.42b	38.88 ± 0.17c
12	42.08 ± 0.61a	41.67 ± 0.32bc	86.88 ± 0.65ab	42.55 ± 0.22b
20	42.28 ± 0.11a	42.21 ± 0.46ab	86.77 ± 0.33ab	44.14 ± 0.41ab
24	41.84 ± 0.10ab	43.91 ± 0.30a	86.90 ± 0.42a	44.23 ± 0.13a

Mean ± standard deviation, similar letters have no significant effect.

Antioxidant activity (%), as presented in the Table 4, showed an increase from 0 to 24 h of fermentation. Kwaw et al.^42^ discovered a relationship between polyphenolic and antioxidant activities. The findings suggested that processing of fermented beverages enhanced the total phenolic content and total flavonoid content, resulting in higher antioxidant activity in the developed product.

### Comparative evaluation of traditionally fermented *Kanji* beverage and reconstituted *Kanji* beverage

The physicochemical parameters were slightly varied in the fermented *Kanji* beverage prepared by reconstituted as compared to the standard traditional fermented *Kanji* beverage as shown in the Table 5. Despite higher pH and less acidification was observed for the reconstituted one when compared to the traditional one, there is no significant differences (*p* > 0.05) observed for pH and titratable acidity.

**Table 5:** Physicochemical characteristics of traditional and LAB fermentation of *Kanji* beverage reconstituted at 10:60 concentration.

Parameters	Traditional *Kanji* beverage	Reconstituted *Kanji* beverage
Titrable acidity (%Lactic acid)	0.92 ± 0.11a	0.87 ± 0.12a
pH	3.94 ± 0.16a	3.70 ± 0.14a
˚Brix	2.90 ± 0.29a	2.70 ± 0.17b
Brix-Acid ratio	3.15 ± 0.21a	3.10 ± 0.19b
Total sugars (mg/mL)	26.98 ± 0.17a	24.57 ± 0.15b
Total reducing sugars (mg/ml)	29.15 ± 0.32a	26.34 ± 0.21b
Antioxidant activity (%)	82.34 ± 0.44b	86.90 ± 0.42a
Total flavonoid content; (mg/100 ml)	42.32 ± 0.46b	43.91 ± 0.30a
Total phenolic content; (mg/100 ml)	40.12 ± 0.21b	41.84 ± 0.10a
Total carotenoids; (mg/100 ml)	46.98 ± 0.11a	44.23 ± 0.13b
Ascorbic acid content; (mg/100 ml)	108 ± 0.12a	98 ± 0.20b

Mean ± standard deviation, similar letters have no significant effect.

The total phenolic content, total flavonoid content and antioxidant activity values of both the traditional and rehydrated *Kanji* beverage are in the same line. Tangüler^43^ found similar results in the shalgam powder production from fermented shalgam (a traditional Turkish beverage). Shalgam powder reconstituted at 3.2% has similar physicochemical properties as in the traditional shalgam beverage in terms of pH, titratable acidity, total solids, polyphenols, flavonoids content and antioxidant capacity. Thus, *Kanji* beverage prepared from reconstitution of RWD-dried powder can be a good alternative with increased shelf-life to obtain functional benefits of *Kanji* beverage throughout the year.

**Figure 3:**
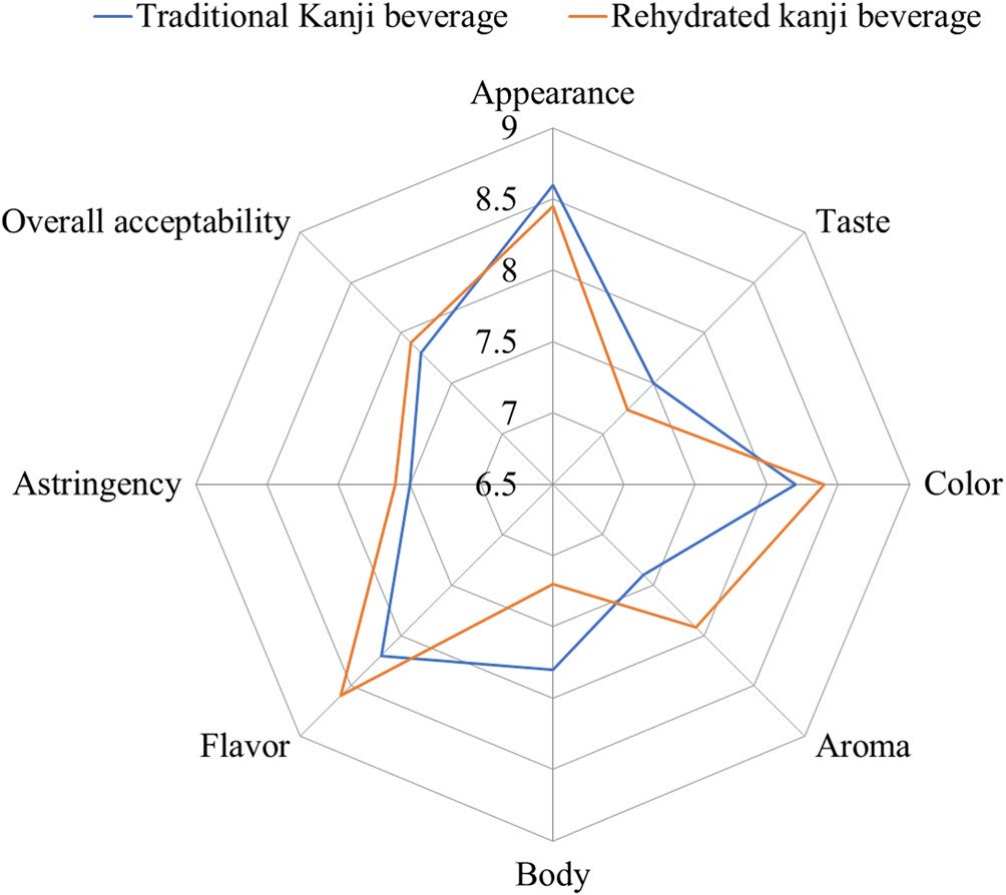
Overall acceptance score of traditional *Kanji* beverage and LAB fermentation of *Kanji* beverage reconstituted at 10:60 concentration.

The mean acceptability scores of reconstituted *Kanji* beverage and conventional beverage are shown in Fig. 3. For the qualities taste and general acceptability, the reconstituted was marginally better accepted (*p* < 0.5) than the traditional. There were no significant changes in colour, astringency, or aroma across the samples (*p* > 0.05). The reconstituted beverage's mean acceptance ratings were about (7.9) on the hedonic scale, which corresponds to "liked very much," showing strong acceptance of the sample, which was also seen for the conventional one. Santos et al.^44^ investigated freeze dried yoghurts and discovered moisture, total solids, and lactic acid percentages of 79.44, 20.86, and 0.92, respectively, as well as pH of 4.37 and diacetyl of 11.13 mg/50 mL in the reconstituted product (5 g/20 °C). These researchers received ratings of 6.67, 6.82, 6.77, and 6.45 on a 9-point hedonic scale for the rehydrated product's appearance, flavour, taste, texture, and overall approval, respectively.

## Conclusion

Plant-based probiotic beverages have been witnessing a rise in demand as well as acceptance in the diet of consumers owing to consumer demand for functional non-dairy products. Fermented black carrot beverage ‘*Kanji*’ is a traditional drink that has been prepared in Indian household since ages. This beverage is highly rich in bioactives and flavonoids, having considerable health benefits to consumers. In this study an effort has been made to prepare a nutrition-rich beverage that can be made available in ready to use format for consumption throughout the year by using advanced drying technologies. The study focused on development and standardization of bioprocess technology for the shelf-stable *Kanji* mix incorporating freeze-dried lactic acid bacterial culture in RW-dried black carrot powder and spices. Black carrot powder was obtained by dipping black carrot in 0% NaOH solution and drying puree at water temperature of 70 °C in RW dryer. The *Kanji* mix was reconstituted with water at concentration of 10:60 and subjected to controlled fermentation for 24 h. The reconstituted beverage was found to be microbiologically safe with acceptability equivalent to traditionally prepared *Kanji* beverage. It was concluded that using this technique similar LAB fermented drinks can be prepared as traditional method of fermentation is not controlled, causing an increase in bacterial count and making the drink unfit for consumption.

## Data Availability

The datasets generated during and/or analysed during the current study are available from the corresponding author on reasonable request.
